# Transactions of the Southern Surgical and Gynecological Association

**Published:** 1890-12

**Authors:** 


TRANSACTIONS OF THE SOUTHERN SURGICAL AND GYNECOLOGICAL ASSOCIATION. Volume II., second session, held at Nashville, Tenn., November 12th, 13th and 14th, 1889, 8vo, pp. xxxvi.—379. Published by the Association at Philadelphia. William J. Dornan, Printer. 1890.

This volume contains the papers read, and the discussions thereon, before this association at its second annual session. It embraces the list of officers and members, the constitution and by-laws of the association, and the minutes of the second annual meeting, together with twenty-seven titles of papers on subjects pertaining to surgery and gynecology. It also contains numerous illustrations that elucidate the text in a capable manner.

It would be difficult to find a more attractive volume of transactions in the work of any society with which we are acquainted. It is most admirably edited by the secretary of the society, Dr. W. E. B. Davis, of Birmingham, Ala., and can be obtained either of him or of the printer, William J. Dornan, 100 North Seventh street, Philadelphia, Pa.

An association of American physicians and surgeons that creates a volume of such excellence during a three days' session, need not fear to offer its work annually for a period that shall embrace the ages.

